# Bioactive Potential and COX-2 Interaction of *Ajuga iva* (L.) Schreb. Hydroalcoholic Extract: Evidence from Experimental and Computational Studies

**DOI:** 10.3390/molecules31030496

**Published:** 2026-01-31

**Authors:** Yousra Boutora, Samira Boussekine, Ouided Benslama, Sabrina Lekmine, Nedjwa Mansouri, Nabil Touzout, Hamza Moussa, Rania Gacem, Najla Hfaiedh, Gema Nieto

**Affiliations:** 1 Laboratory of Bioactive Molecules and Applications, Department of Applied Biology, Faculty of Exact Sciences and Natural and Life Sciences, Echahid Cheikh Larbi Tebessi University, Tebessa 12000, Algeria; yousra.boutoura@univ-tebessa.dz (Y.B.); boussekinesa@yahoo.fr (S.B.); rania.gace@univ-tebessa.dz (R.G.); 2 Department of Natural and Life Sciences, Faculty of Exact Sciences and Natural and Life Sciences, Larbi Ben M’Hidi University, Oum El Bouaghi 04000, Algeria; benslama.wided@hotmail.fr (O.B.);; 3 Laboratory of Biotechnology, Water, Environment and Health (LBWEH), University of Abbes Laghrour, Khenchela 40000, Algeria; 4 Higher National School of Forest, Khenchela 40000, Algeria; 5 Department of Agronomy, Faculty of Sciences, Pole Urban Ouzera, University of Medea, Medea 26000, Algeria; 6 Laboratory Materials and Environment, University Yahia Fares of Medea, Urban Center, Medea 26000, Algeria; 7 Laboratoire de Gestion et Valorisation des Ressources Naturelles et Assurance Qualité (LGVRNAQ), Faculté des Sciences de la Nature et de la Vie et des Sciences de la Terre, Université de Bouira, Bouira 10000, Algeria; hamza.moussa49@gmail.com; 8 Département des Sciences Biologiques, Faculté des Sciences de la Nature et de la Vie et des Sciences de la Terre, Université de Bouira, Bouira 10000, Algeria; 9 Laboratory of Biotechnology and Biomonitoring of the Environment and Oasis Ecosystems (LBBEEO), Located at the Faculty of Sciences of Gafsa, University of Gafsa, Gafsa 2112, Tunisia; najlaharrathi@yahoo.fr; 10 Department of Food Technology, Nutrition and Food Science, Veterinary Faculty, University of Murcia, Regional Campus of International Excellence “Campus Mare Nostrum”, 30100 Espinardo, Murcia, Spain

**Keywords:** *Ajuga iva*, hydroalcoholic extract, anti-inflammatory, analgesic, antipyretic

## Abstract

*Ajuga iva* (L.) Schreb. is traditionally used in North African ethnomedicine for the management of inflammation, pain, and fever. The present study aimed to characterize the phytochemical profile of the hydroalcoholic extract of its aerial parts and to evaluate its anti-inflammatory, analgesic, and antipyretic activities using established in vivo models. Preliminary phytochemical screening confirmed the presence of major classes of secondary metabolites, including polyphenols, flavonoids, tannins, and glycosidic compounds. Quantitative assays revealed appreciable levels of total phenolics (26.3 ± 1.2 mg GAE/g extract) and flavonoids (13.5 ± 0.9 mg QE/g extract). In vivo pharmacological evaluation demonstrated significant biological activities, with the highest tested dose (400 mg/kg) producing a marked inhibition of carrageenan-induced paw edema (44.9%), comparable to acetylsalicylic acid. At the same dose, the extract showed pronounced analgesic activity in the acetic acid-induced writhing test, with an inhibition rate of 64.2%, and a significant antipyretic effect in the brewer’s yeast-induced fever model, as evidenced by a reduction in rectal temperature. In parallel, molecular docking was employed as an exploratory, hypothesis-generating in silico approach to investigate potential interactions between selected phenolic constituents identified in *A. iva* and cyclooxygenase-2 (COX-2). Several compounds, including rosmarinic acid, rutin, and apigenin-7-O-glucoside, displayed favorable predicted binding affinities and interactions with key residues of the COX-2 active site. It should be emphasized that molecular docking was used solely as a hypothesis-generating in silico tool and does not constitute direct biochemical evidence of COX-2 inhibition. Overall, these findings indicate that the hydroalcoholic extract of *Ajuga iva* exhibits notable anti-inflammatory, analgesic, and antipyretic activities in vivo. The in silico docking results provide supportive, predictive molecular insights that may help rationalize the observed bioactivities and encourage further biochemical and mechanistic investigations into this traditionally used medicinal plant.

## 1. Introduction

Medicinal plants continue to play a crucial role in the discovery of novel therapeutic agents, due to their richness in structurally diverse bioactive metabolites, including alkaloids, flavonoids, terpenoids, phenolics, polyphenols, and essential oils [1]. These naturally occurring compounds exhibit a wide range of pharmacological properties, such as antibacterial, antifungal, antioxidant, anti-inflammatory, and anticancer activities [2,3]. Among medicinal plants, *Ajuga iva* L. has been traditionally used for the treatment of various human disorders [4,5].

*A. iva* Schreb. is a perennial herbaceous species belonging to the Lamiaceae family and is native to the Mediterranean region [6,7]. In North African traditional medicine, it is commonly known as “Chandgoura” [8]. The aerial parts of the plant, including leaves and flowering tops, are traditionally prepared as decoctions or infusions to treat a variety of ailments, such as inflammatory disorders, digestive disturbances, diabetes, infections, pain, and rheumatism. Morphologically, *Ajuga iva* is a low-growing plant, reaching approximately 20 cm in height, with compact and dense leaves. Its inflorescences consist of solitary flowers developing in the leaf axils, typically flowering between May and June, while its fruits are reticulated nutlets, a characteristic feature of the species [8,9].

In North African ethnomedicine, particularly in Algeria, Tunisia, and Morocco, *A. iva* has been widely used to manage gastrointestinal disorders, hypertension, hyperglycemia, diabetes, alopecia, and pain-related conditions. Phytochemical investigations have revealed that the plant is rich in bioactive constituents, including flavonoids, phenolic acids, iridoids, tannins, phytoecdysteroids, terpenoids, organic acids, vitamins, minerals, glycosides, and fatty acid methyl esters [8]. This chemical diversity provides a strong scientific rationale for its traditional therapeutic applications. Inflammation is a complex biological response that plays a vital role in host defense; however, its dysregulation is closely associated with the development of chronic diseases, including autoimmune disorders and cancer. Conventional anti-inflammatory agents, such as glucocorticoids and non-steroidal anti-inflammatory drugs, exert their effects primarily through the inhibition of cyclooxygenase (COX) enzymes and other mediators. Despite their clinical efficacy, the long-term use of these drugs is often limited by serious adverse effects, including gastrointestinal, renal, and cardiovascular toxicity [10].

Modern discovery strategies increasingly integrate experimental and computational approaches. In silico molecular docking, in particular, allows rapid prediction of ligand–target interactions and provides valuable insight into potential mechanisms of action [11,12], especially regarding the inhibition of inflammation-related enzymes such as cyclooxygenase-2 (COX-2), a key therapeutic target. The combination of computational modeling with in vivo validation enhances the reliability of natural product-based drug discovery by linking chemical composition to pharmacological activity.

Overall, this study adds value by integrating in vivo biological evaluations with LC–MS-based phytochemical profiling and molecular docking analysis, thereby providing complementary evidence supporting the pharmacological relevance of the regional *Ajuga iva* chemotype collected from the Tebessa region of Algeria. The observed anti-inflammatory, analgesic, and antipyretic activities are consistent with, and comparable to, previously reported findings. Furthermore, molecular docking results suggest potential interaction between the identified bioactive compounds and cyclooxygenase-2 (COX-2), offering a predictive mechanistic rationale for the observed biological effects. However, these in silico findings remain exploratory in nature and require further biochemical and molecular validation to confirm the involvement of COX-2 inhibition in the therapeutic potential of this traditionally valued medicinal plant.

## 2. Materials and Methods

### 2.1. Plant Material Collection and Preparation of Extract

The aerial parts of *Ajuga iva* were collected from the Tebessa region, Algeria. The botanical identity of the plant was confirmed by Dr. Hioun Soraya, a plant taxonomist at University of Tebessa, Algeria. Due to the unavailability of a local herbarium, a voucher specimen will be deposited at a recognized herbarium and assigned a voucher ID upon acceptance of the manuscript. The hydroalcoholic extract was prepared following standard procedures: 100 g of dried plant material were macerated in 1 L of 70% methanol for 24 h with occasional shaking. The mixture was filtered through Whatman No. 1 filter paper, and the solvent was evaporated under reduced pressure at 40 °C using a rotary evaporator. The obtained extract was then dried and stored at 4 °C until analysis. Extraction yield was calculated as the weight of the dried extract relative to the starting plant material, giving a yield of 14.08% *w*/*w*.

### 2.2. Phytochemical Screening

Various classical colorimetric tests were employed to detect the main groups of bioactive metabolites: ferric chloride test for tannins and polyphenols, Shinoda test for flavonoids, foam test for saponins, Liebermann–Burchard reaction for terpenes and sterols, Borntrager’s test for glycosides, and Mayer’s reagent for alkaloids [13].

### 2.3. Determination of Total Phenolic and Flavonoid Contents

The Folin–Ciocalteu procedure as used to measure total phenolic content (TPC). In brief, 200 µL of the extract was mixed with 600 µL of diluted Folin reagent (10%). After 3 min, 800 µL of Na_2_CO_3_ solution (7.5%) was added. The mixture was kept in the dark for 30 min, and absorbance value was recorded at 765 nm, the gallic acid equivalents (mg GAE/g extract) have been used to express the results [14].

The amount of total flavonoid (TFC) was measured using the aluminum chloride colorimetric technique. A mixture of 0.5 mL extract with 2 mL distilled water was prepared, followed by addition of 0.15 mL NaNO_2_ (5%). After 5 min, 0.15 mL AlCl_3_ (10%) was added. Following the addition 1 mL of NaOH (1 M) was introduced after six minutes later, and the absorbance was measured at 430 nm. Results were expressed as mg quercetin equivalents per gram (mg QE/g) [15].

### 2.4. LC-MS Analysis of Phenolic Compounds

The identification and quantification of phenolic compounds were performed using LC-MS techniques. Analyses were carried out on a Shimadzu UFLC XR system 5.91(Kyoto, Japan) equipped with a SIL-20AXR auto-sampler, (Shimadzu Corporation, Kyoto, Japan) CTO-20 AC column oven, LC-20ADXR binary pump, and a quadrupole 2020 detector. Separation was achieved on an Inertsil ODS-4 C18 column (3 µm, 150 × 3.0 mm i.d.) maintained at 40 °C, with an injection volume of 20 µL and a flow rate of 0.5 mL/min. Mobile phases consisted of A (95% H_2_O + 5% MeOH + 0.2% acetic acid) and B (50% ACN + 50% H_2_O + 0.2% acetic acid), applied with a linear gradient: 0.01–14 min, 10–20% B; 14–27 min, 20–55% B; 27–37 min, 55–100% B; 37–45 min, 100% B; 45–50 min, 10% B. The desolvation line temperature was set at 275 °C, nebulizing gas flow at 1.50 L/min, drying gas at 15.00 L/min, and heat block temperature at 450 °C. LC-ESI(–) MS spectra [M–H]^−^ were acquired using LabSolutions LCMS version 5.91 (Shimadzu Corporation, Kyoto, Japan), and compounds were identified by comparison of retention times and mass spectra with authentic standards. All analyses were performed in triplicate to ensure reproducibility [16].

Calibration curves were constructed from authentic standards across six concentration levels covering the analytical range. Peak areas were plotted against nominal concentrations, and weighted linear regression (1/x) was applied. Linearity was excellent (R^2^ = 0.991–0.999), with back-calculated concentrations within ±15% of nominal values (±20% at the LLOQ). Method validation included accuracy, precision, sensitivity, and recovery. Intra-day reproducibility showed RSD% consistently below 5%, while inter-day reproducibility was below 7%. Limits of detection (LOD) ranged between 0.05 and 0.2 µg/mL, and limits of quantification (LOQ) between 0.15 and 0.5 µg/mL. Recovery rates for spiked samples were between 92% and 105%, confirming accuracy and robustness.

Carryover was assessed by injecting a blank matrix sample immediately after the highest calibration standard and high-concentration samples. Chromatograms of these blanks were examined for residual analyte peaks, with acceptance criteria requiring that any carryover signal not exceed 20% of the LLOQ response. No residual peaks were observed. Instrumental conditions were optimized to prevent saturation, including adjustment of injection volume, use of needle wash solutions, and gradient modifications to ensure complete elution. Representative chromatograms of high-concentration samples and subsequent blanks are provided in the Supplementary Materials to illustrate the absence of carryover.

Together, these validation parameters and carryover assessments demonstrate that the method is sensitive, accurate, and reproducible, and that chromatographic saturation does not compromise trace-level quantification.

### 2.5. Animal

Adult animals (Wistar rats and Swiss albino mice) were obtained from the Pasteur Institute, Algeria, a certified and accredited public institution for laboratory animal breeding and supply. They were acclimated for one week under standard laboratory conditions, including a controlled temperature of 22 ± 2 °C, a 12-h light/dark cycle, and free access to food and water. All experimental procedures were conducted in accordance with internationally accepted guidelines for the care and use of laboratory animals (ARRIVE guidelines and the principles of the 3Rs) and were approved by the Institutional Animal Care and Use Committee (IACUC) under registration number PI-AL-2025-07. Every effort was made to minimize animal suffering, and the minimum number of animals necessary to achieve statistically valid results was used. A post-hoc power analysis indicated that the chosen sample size (*n* = 5 for most experiments) provides >80% power to detect significant differences in anti-inflammatory and analgesic responses.

### 2.6. Anti-Inflammatory Activity

The anti-inflammatory potential was examined using the λ-carrageenan-induced paw edema model in rats following Elion Itou et al. (2017) [17], with slight adjustments. Rats were randomly assigned into five groups (*n* = 5): a vehicle control group receiving distilled water (T−), three groups treated orally with the deferent doses of extract (EHA), dissolved in distilled water at doses (100, 200 and 400 mg/kg body weight) and a reference drug group (T+) receiving acetylsalicylic acid (75 mg/kg).

Paw volume was monitored before injection and every hour for five hours using the mercury displacement technique [18].

### 2.7. Analgesic Activity

According to Koster et al. (1959) [19], after a fasting period of 18 h five groups of mice were formed (*n* = 5). The vehicle control group (T−) were treated with saline solution, and the other groups were delivered the extract at doses (100, 200 and 400 mg/kg body weight), and a reference drug group (T+) receiving acetylsalicylic acid (75 mg/kg) by oral gavage using a stainless-steel gavage needle (18G, rounded tip). One hour later, 0.1 mL of 3% acetic acid was injected intraperitoneally, and the number of writhes was counted over 30 min.

### 2.8. Antipyretic Activity

Antipyretic activity was tested using the brewer’s yeast-induced fever model following. Mice were fasted for 18 h, then injected subcutaneously with 10 mL/kg of yeast aqueous suspension (*Saccharomyces cerevisiae*, 15%) to induce hyperthermia. Four groups (*n* = 5) were used: vehicle control group receiving distilled water (T−), two groups receiving extract at doses (200 and 400 mg/kg body weight), and reference drug group (T+) receiving acetylsalicylic acid (75 mg/kg). Rectal temperature was measured hourly for five hours using a digital thermometer [20,21].

### 2.9. In Silico Evaluation

#### 2.9.1. Protein Preparation for Docking

The crystal structure of COX-2 (PDB ID: 1CX2) was downloaded from the Protein Data Bank. All water molecules and heteroatoms were removed, while the co-crystallized ligand (S58) was kept to identify the active site pocket. Polar hydrogens and Kollman charges were added using AutoDock Tools.

#### 2.9.2. Ligand Preparation

Phenolic compounds previously reported in *A. iva* were obtained from PubChem in SDF format. Their structures were minimized and converted into. PDBQT format through AutoDock Tools. The reference ligand S58 was prepared similarly for redocking.

#### 2.9.3. Docking Procedure

Molecular docking studies were conducted with AutoDock version 4.2.6 [22]. The grid box was centered on the coordinates of the co-crystallized ligand to cover the COX-2 catalytic region. The Lamarckian Genetic Algorithm (LGA) was applied with default parameters, generating several poses for each compound, ranked by estimated binding free energy (kcal/mol) [16].

#### 2.9.4. Docking Validation

To ensure docking accuracy, the ligand S58 was redocked into the active site. The resulting pose presented an RMSD of 0.557 Å relative to the experimental structure, indicating good reliability (RMSD < 2 Å).

#### 2.9.5. Visualization and Interaction Analysis

Analysis of hydrogen bonding, hydrophobic interaction, and electrostatic forces was performed using Discovery Studio Visualizer (version 21.1.0.20298). Binding energies and interaction patterns were examined to identify residues contributing to ligand stabilization.

### 2.10. Statistical Analysis

All graphical representations and statistical analyses were performed using GraphPad Prism version 10 (GraphPad Software 10, San Diego, CA, USA). Experimental data are expressed as mean ± SD. Statistical comparisons among groups were conducted using one-way analysis of variance (ANOVA), followed by Dunnett’s post hoc test for multiple comparisons against the vehicle control group.

The selected group sizes (*n* = 5 per group) are consistent with those commonly employed in preliminary in vivo pharmacological studies using carrageenan-induced paw edema, acetic acid-induced writhing, and yeast-induced pyrexia models. Although no a priori formal power calculation was performed, statistically significant differences were observed between treated and vehicle groups under the present experimental conditions.

Differences were considered statistically significant at *p* < 0.05, with significance levels indicated as * *p* < 0.05, ** *p* < 0.01, and *** *p* < 0.001.

## 3. Results

### 3.1. Phytochemical Screening of the Hydroalcoholic Extract of A. iva

The qualitative phytochemical analysis of the hydroalcoholic extract of *A. iva* demonstrated the presence of several major classes of secondary metabolites (Table 1). The extract was particularly rich in tannins and polyphenols (+++), indicating a high content of phenolic constituents known for their antioxidant and therapeutic potential. Moderate levels (++) of flavonoids, glycosides, sterols, and terpenes were also observed, suggesting additional biological relevance. A weak reaction (+) for alkaloids was recorded, while saponins were absent (−). These findings confirm the chemical diversity of the extract and highlight its potential pharmacological importance.

### 3.2. Overall Phenolic and Flavonoid Levels

The total amount of phenolics and flavonoids of the hydroalcoholic extract of *A iva* (L.) Schreb. were determined by colorimetric assays (Table 2). The total phenolic and flavonoid contents of the hydroalcoholic extract of *A. iva* (L.) Schreb. were quantified using standard colorimetric assays, and the results are summarized in Table 2. The extract exhibited a Total Phenolic Content (TPC) of 26.3 ± 1.2 mg GAE/g dry extract, based on a calibration curve obtained with gallic acid (R^2^ = 0.9996). The Total Flavonoid Content (TFC) reached 13.5 ± 0.9 mg QE/g dry extract, calculated using a quercetin standard (R^2^ = 0.9969). These values indicate that the hydroalcoholic extract is a rich source of phenolic and flavonoid compounds, which may contribute to its observed biological activities.

### 3.3. LC–MS/MS Identification of Phenolic and Flavonoid Compounds

The liquid chromatography–mass spectrometry (LC-MS) analysis of the hydroalcoholic extract of *A iva* (L.) Schreb. revealed the presence of a wide range of phenolic acids and flavonoids with significant pharmacological potential (Table 3). Among the identified compounds, kaempferol (0.3560 mg/g), apigenin (0.9980 mg/g), and rutin (0.0963 mg/g) were the most abundant, suggesting that these flavonoids are the major constituents contributing to the antioxidant and therapeutic effects of the extract. Other notable compounds included naringenin (0.0368 mg/g), the 7-O-glucosilated form of apigenin (0.0738 mg/g), and the 7-O-glucosylated form of luteolin (0.0123 mg/g), known for their anti-inflammatory and enzyme-inhibitory activities. Phenolic acids such as quinic (0.1810 mg/g), p-coumaric (0.0165 mg/g), and protocatechuic (0.0097 mg/g) acids were also detected, reinforcing the extract’s richness in polyphenolic structures. Minor compounds like rosmarinic, syringic, and *trans*-cinnamic acids were found in lower concentrations but likely act synergistically with the dominant flavonoids. Overall, these findings demonstrate that *A. iva* is a valuable source of bioactive secondary metabolites with potential health-promoting properties.

### 3.4. Anti-Inflammatory Activity

**Figure 1:**
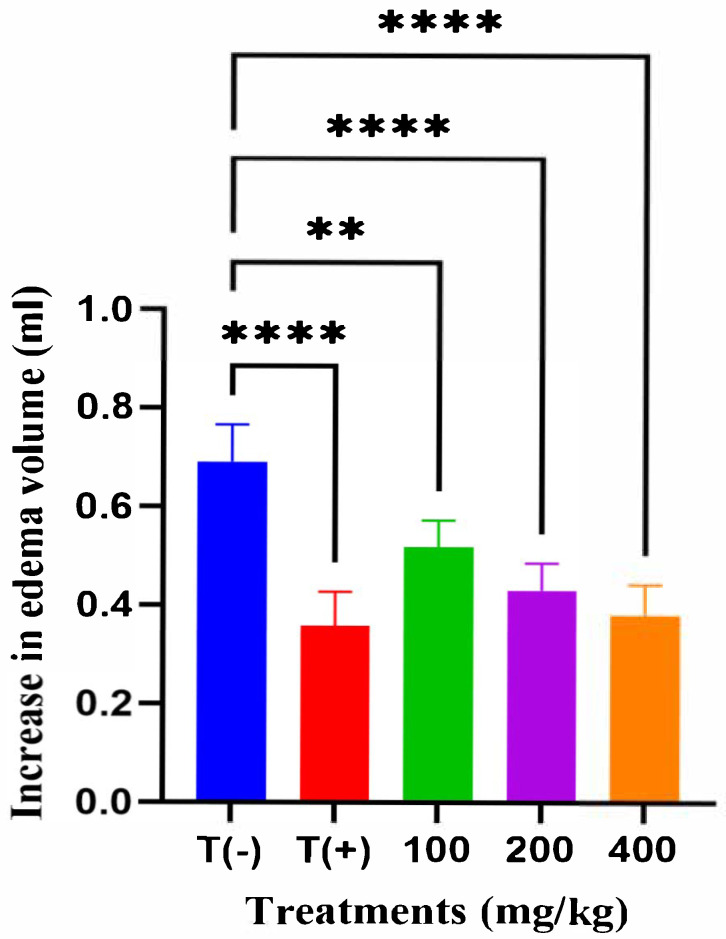
Effect of *A. iva* hydroalcoholic extract on carrageenan-induced paw edema in rats at the 3rd hour ** *p* < 0.01, **** *p* < 0.0001 vs. vehicle control (T−).

At the 3rd hour after carrageenan injection, the vehicle control group (T−), treated with distilled water, showed the highest edema volume (0.69 ± 0.08 mL), confirming successful induction of inflammation. In contrast, the reference drug group (T+), treated with acetylsalicylic acid (75 mg/kg), exhibited a marked decrease in paw edema (0.36 ± 0.07 mL). Administration of *A. iva* extract at doses of 100, 200, and 400 mg/kg produced a progressive, dose-dependent reduction in paw edema (0.52 ± 0.05 mL, 0.43 ± 0.05 mL, and 0.38 ± 0.06 mL, respectively) (Figure 1). Statistical analysis (ANOVA, *p* < 0.0001) followed by Dunnett’s test showed that the 200 and 400 mg/kg doses produced a very highly significant reduction compared with the vehicle control (T−), and the 100 mg/kg dose showed a significant effect (*p* < 0.01).

### 3.5. Analgesic Activity

**Figure 2:**
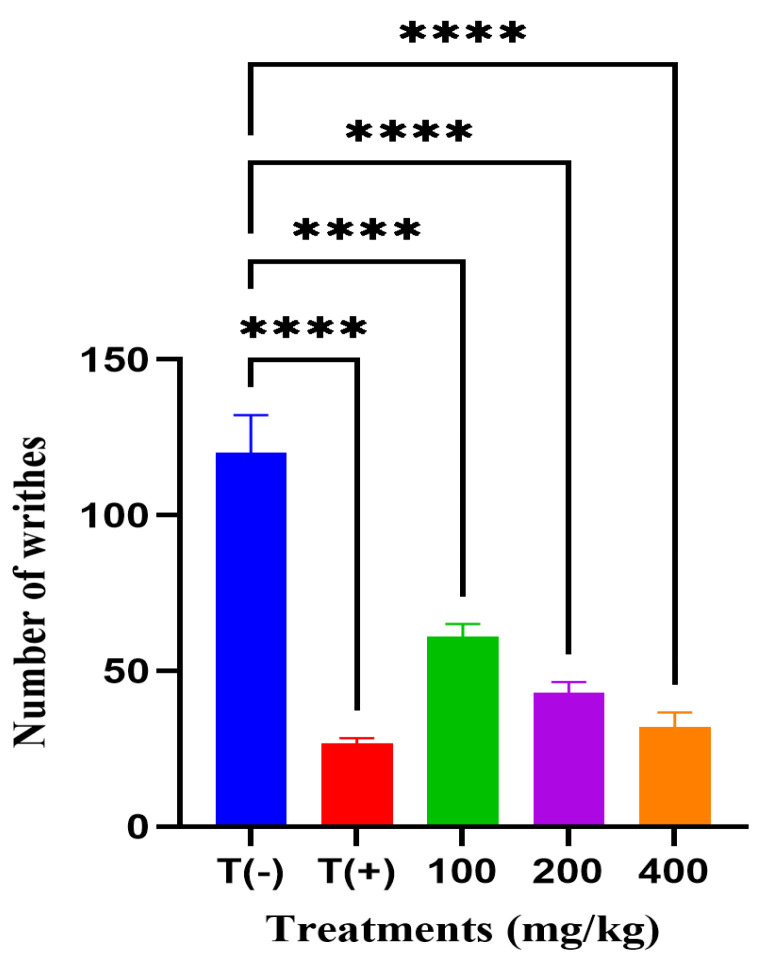
Effect of *A iva* hydroalcoholic extract on analgesic activity as measured by the number of writhes in mice **** *p* < 0.0001 vs. vehicle control (T−).

Pain-relieving effect of the hydroalcoholic extract of *A. iva* was assessed using the acetic acid-induced writhing test in mice. The vehicle control group (T−), treated with distilled water, exhibited the highest number of writhes (120 ± 10.86), confirming the successful induction of nociception. In contrast, the reference drug group (T+), administered acetylsalicylic acid (75 mg/kg), exhibited a notable decrease to 27 ± 1.35 writhes. Treatment with *A. iva* extract at 100, 200, and 400 mg/kg produced a writhing inhibition showing a dose-dependent increase, yielding 61 ± 3.31, 43 ± 2.82, and 32 ± 4.04 writhes, respectively (Figure 2). All experimental groups showed a statistically highly significant effect relative to the vehicle control, with all comparisons reaching **** (*p* < 0.0001).

### 3.6. Antipyretic Activity

**Figure 3:**
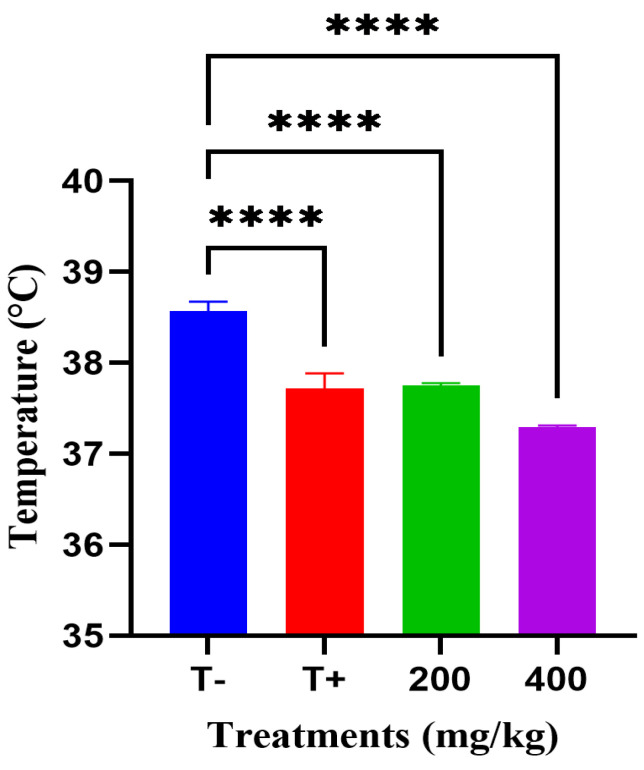
Effect of *A. iva* hydroalcoholic extract in the brewer’s yeast induced pyrexia model in rats. **** *p* < 0.0001 vs. vehicle control (T−).

Fever-reducing effect of the hydroalcoholic extract of *A. iva* was assessed (Figure 3). The vehicle control group (T−), treated with distilled water, exhibited the highest mean rectal temperature (38.57 °C), confirming successful induction of fever. In contrast, the reference drug group (T+), treated with acetylsalicylic acid (75 mg/kg),), showed a significant reduction in body temperature to 37.72 °C (ANOVA, *p* < 0.0001). Administration of *A. iva* extract at 200 mg/kg produced a comparable reduction (37.74 °C), and 400 mg/kg reduced body temperature to 37.28 °C. All experimental groups showed a statistically highly significant effect relative to the vehicle control, with all comparisons reaching **** (*p* < 0.0001).

### 3.7. In Silico Evaluation

The docking study aimed to assess the potential inhibitory binding of *A. iva* phenolic compounds with the cyclooxygenase-2 (COX-2) enzyme (PDB ID: 1CX2), which plays a key role in the inflammatory response. All the identified compounds from the hydroalcoholic extract were analyzed, and their binding affinities were found to range from −8.8 kcal·mol^−1^ for rosmarinic acid to −5.3 kcal·mol^−1^ for the smallest molecule, quinic acid.

The best ten compounds, based on their docking scores, are presented in Table 4. These compounds exhibited predicted binding energies ranging from −8.8 to −6.2 kcal·mol^−1^, indicating a favorable theoretical accommodation within the COX-2 active site at a structure-based, predictive level. These docking scores should be interpreted as indicative of potential binding modes rather than direct evidence of inhibitory activity. Two-dimensional representations of the best-ranked docked complexes are provided in Figures S1 and S2, highlighting the main predicted ligand–residue interactions within the COX-2 binding pocket.

Among the tested compounds, rosmarinic acid displayed the most favorable interaction (–8.8 kcal/mol), forming multiple hydrogen bonds with key residues such as Gln192, Ser353, Tyr355, and Tyr385, and additional hydrophobic contacts with Val523, Ser353, and Gly526, important residues known to stabilize inhibitors in the COX-2 catalytic site.

Rutin and apigenin-7-O-glucoside also exhibited strong binding energies (–8.7 and –8.5 kcal/mol, respectively). According to molecular docking predictions, rutin was predicted to form an extensive hydrogen-bonding network involving Val523, Ser353, Phe518, Gln192, and His90. Similarly, apigenin-7-O-glucoside was predicted to establish multiple hydrogen-bonding and hydrophobic contacts with Tyr385, Gln192, Leu352, and Ser353. These interactions illustrate the plausible binding orientations of the ligands within the COX-2 binding pocket, as visualized in Figure S1 Compounds such as acacetin (–8.3 kcal/mol) and luteolin-7-O-glucoside (–7.6 kcal/mol) also displayed favorable docking scores, engaging with catalytic residues His90, Tyr355, Arg120, and Ser530, which are essential for enzyme inhibition.

Moderate interactions were observed for naringenin (–7.5 kcal/mol), kaempferol (–7.1 kcal/mol), apigenin (–6.7 kcal/mol), luteolin (–6.5 kcal/mol), and quercetin (–6.2 kcal/mol), forming stable hydrogen bonds and hydrophobic interactions within the COX-2 binding pocket (Figure S2).

## 4. Discussion

Phytochemical screening of the hydroalcoholic extract of *Ajuga iva* revealed the presence of multiple classes of secondary metabolites, including polyphenols, flavonoids, tannins, alkaloids, sterols, and terpenes. These findings are consistent with previous reports on *A. iva* (Saidi et al. 2023., Makni 2013), confirming the chemical richness of this species and supporting its pharmacological relevance [23,24]. Natural plant extracts such as *A. iva* typically contain diverse bioactive compounds may act in a complementary or synergistic manner, leading to complex pharmacological effects rather than single-compound activity [25,26,27].

LC–MS/MS analysis further demonstrated that the extract is particularly rich in flavonoids and phenolic acids, compound classes widely associated with antioxidant, anti-inflammatory, and antipyretic activities [28]. Importantly, all pharmacological evaluations in the present study were performed using the crude hydroalcoholic extract. Therefore, the observed biological effects should be interpreted at the extract level and are more plausibly explained by the combined and potentially synergistic action of multiple phytochemical classes rather than by isolated individual compounds [29,30]. The phytochemical composition of *A. iva* further supports its pharmacological relevance, as these compounds have been previously implicated in anti-inflammatory, analgesic, antimicrobial antioxidant activities, with applications extending to the food and cosmetic industries and involvement in nociceptive pathways [31,32,33].

Quantitative analysis showed that the hydroalcoholic extract of *A. iva* contained substantial amounts of total polyphenols (26.3 ± 1.2 mg GAE/g dry extract) and total flavonoids (13.5 ± 0.9 mg QE/g dry extract). These values are higher than those reported by Mohamed et al. [24] for a methanolic extract of *A. iva* (25.69 ± 2.6 mg GAE/g and 3.00 ± 0.3 mg QE/g extract), lower than the total phenolic content of an ethanolic extract reported by Bouyahya et al. [34] (49.75 ± 2.08 mg GAE/g), and comparable to the values reported by Medjeldi et al. [35] (28.3 ± 1.12 mg GAE/g). Total flavonoid contents reported for other Ajuga species, such as A. chamaepitys (24.72 ± 0.24 mg QE/g dry extract) [36] further highlight interspecific and extraction-related variability.

Inflammation, pain, and fever are closely interconnected physiological defense responses that protect tissues against damage and infection; however, when dysregulated, they contribute to pathological conditions, including arthritis, diabetes, neurodegenerative disorders, and autoimmune diseases [37,38]. Consequently, plant-derived bioactive molecules with comparable therapeutic efficacy but fewer adverse effects have attracted increasing scientific and clinical interest [39,40].

In the present study, the hydroalcoholic extract of *A. iva* exhibited significant anti-inflammatory, analgesic, and antipyretic activities, all of which followed a clear dose-dependent pattern. In the carrageenan-induced paw edema model, the extract significantly inhibited inflammation in a dose-dependent manner, suggesting modulation of inflammatory mediators involved in both early and late phases of the inflammatory response [41,42]. This pattern consistent dose–response relationship strengthens the pharmacological interpretation of the results.

Similarly, the extract produced a marked, dose-dependent reduction in acetic acid-induced writhing, indicative of peripheral analgesic activity potentially related to the modulation of inflammatory mediators such as prostaglandins and bradykinins [43]. The antipyretic effect observed in the brewer’s yeast-induced fever model also followed a dose-dependent trend, with significant reduction in elevated body temperature, suggesting possible interference with fever-mediating pathways involving prostaglandin E_2_ and pro-inflammatory cytokines [43].

Recent computational studies highlighting the multitarget therapeutic potential of natural compounds, such as silymarin, further support the relevance of plant-derived bioactive metabolites in pharmacological research [44,45,46,47,48]. Cyclooxygenase-2 (COX-2) is widely recognized as a central molecular target in anti-inflammatory drug discovery due to its inducible expression during inflammation conditions and its role in catalyzing the conversion of arachidonic acid into pro-inflammatory prostaglandins that mediate pain, fever, and inflammation [49,50]. Structurally, COX-2 possesses a larger and more flexible active site than COX-1, allowing selective accommodation of bulkier ligands and enabling the development of COX-2-preferential inhibitors with improved safety profiles [51,52]. Within this context, molecular docking represents a valuable computational tool for exploring potential ligand–target recognition patterns at the structural level.

The novelty of the present work lies in the integration of LC–MS/MS-based phytochemical profiling with structure-based molecular docking applied to a region-specific *Ajuga iva* chemotype collected from the Tebessa region of Algeria. This combined approach provides compound-level mechanistic hypotheses that complement the extract-level in vivo pharmacological findings.

Molecular docking was employed as a hypothesis-generating, structure-based approach to explore potential interactions between phenolic constituents identified in *Ajuga iva* and the COX-2 active site. Several compounds displayed favorable predicted binding energies (–8.8 to –6.2 kcal·mol^−1^), for the top-ranked ligands. while the co-crystallized reference ligand (S58) exhibited a predicted binding energy of –9.4 kcal·mol^−1^. Among the screened compounds, rosmarinic acid, rutin, and apigenin-7-O-glucoside showed the most favorable docking scores, suggesting enhanced accommodation within the COX-2 binding pocket.

Analysis of the predicted binding modes revealed recurrent interaction with amino acid residues commonly involved in COX-2 ligand recognition, including Arg120 and Tyr355 at the entrance of the catalytic channel. were frequently engaged in hydrogen-bonding or electrostatic interactions across several docked complexes. These residues have been widely reported in structural and computational studies as recurrent interaction sites involved in ligand positioning within the COX channel [51]. In the present docking models, rosmarinic acid was predicted to form an electrostatic interaction with Arg120, while other ligands, including luteolin-7-O-glucoside, naringenin, and quercetin, were also predicted to interact with this residue across several docked complexes.

Additional predicted interactions involved residues such as Gln192, Ser353, and Tyr385. For example, rosmarinic acid was predicted to form hydrogen-bond interactions with Ser353 and Tyr385 in the docking models. His90, located near the entrance of the active site, was also predicted to interact with several ligands, including acacetin, apigenin-7-O-glucoside, and kaempferol. A network of hydrophobic contacts involving Leu352, Val349, Ala527, Phe518, Gly526, and Trp387 was observed, suggesting possible van der Waals and π–π interactions.

These predicted interactions are generally in agreement with residues previously reported in computational studies of COX-2 ligand recognition, including Arg120, Tyr355, Gln192, Ser530, and surrounding hydrophobic residues [52]. The overlap between residues identified in the present docking analysis and those reported in the literature supports the structural plausibility of the predicted binding modes.

Importantly, molecular docking does not constitute experimental evidence of enzymatic inhibition. In this work, docking was used solely as a structure-based, hypothesis-generating approach to explore potential interactions between phenolic constituents of *Ajuga iva* and COX-2. No direct COX-2 enzymatic inhibition or prostaglandin quantification assays were performed, and the docking results should therefore be interpreted with caution. Therefore, the computational results should be interpreted with caution and considered as a structural rationale to guide future experimental investigations.

Finally, it should be emphasized that all pharmacological evaluations were conducted using the crude hydroalcoholic extract of *Ajuga iva,* and the observed biological effects cannot be directly attributed to individual phytochemicals. The overall pharmacological activity is more plausibly explained by the combined and potentially synergistic effects of multiple constituents rather than by a single dominant molecule.

A limitation of the present study is the relatively small sample size used in the in vivo experiments, which may reduce statistical power and limit the detection of subtle effects. Future studies employing larger cohorts and power-based experimental designs will be necessary to further substantiate these findings.

## 5. Conclusions

This study demonstrated that the hydroalcoholic extract of the aerial parts of *Ajuga iva* exhibits significant analgesic, antipyretic, and anti-inflammatory activities. These effects are likely associated with its rich phytochemical composition, particularly its high content of polyphenols and flavonoids. Molecular docking was used as an exploratory, hypothesis-generating in silico approach, suggesting potential interactions between selected bioactive metabolites and the COX-2 active site; however, these computational findings do not constitute direct evidence of enzymatic inhibition. Notably, the present study highlights a distinct regional chemotype of *Ajuga iva,* which may contribute to its unique pharmacological profile and provides added value compared with previously reported populations. Overall, the results confirm the pharmacological potential of *Ajuga iva* at the extract level and support its traditional use in the management of inflammation, pain, and fever, while underscoring the need for further studies to isolate active constituents and elucidate their precise mechanisms of action.

## Figures and Tables

**Table 1:** Qualitative phytochemical profile of the hydroalcoholic extract of *A. iva*.

Phytochemical Class	Observation
Tannins	+++
Polyphenols	+++
Flavonoids	++
Saponins	−
Alkaloids	+
Glycosides	++
Sterols	++
Terpenes	++

**Table 2:** Total phenolic and flavonoid contents of the hydroalcoholic extract of *A iva* (L.) Schreb.

Parameter	Standard Used	Calibration Equation	R^2^	Content (mg/g Dry Extract)	Unit Equivalent
Total Phenolic Content (TPC)	Gallic acid	y = 0.0095x + 0.012	0.9996	26.3 ± 1.2	mg GAE/g DE
Total Flavonoid Content (TFC)	Quercetin	y = 0.0072x + 0.010	0.9969	13.5 ± 0.9	mg QE/g DE

**Table 3:** Phenolic and flavonoid molecule classes of *A iva* extract identified by the liquid chromatography–mass spectrometry (LC-MS).

No.	Compound Designation	RT (min)	[M−H]^−^ (*m*/*z*)	Concentration (mg/g)
1	Quinic acid	1.935	191.00	0.1810
2	Protocatechuic acid	6.882	153.00	0.0097
3	Syringic acid	16.462	197.00	0.0010
4	p-Coumaric acid	21.028	163.00	0.0165
5	trans-Ferulic acid	23.322	193.00	0.0040
6	Rutin	24.750	609.00	0.0960
7	Luteolin-7-O-glucoside	25.387	447.00	0.0123
8	Apigenin-7-O-glucoside	27.657	431.00	0.0738
9	Rosmarinic acid	27.313	359.00	0.0020
10	Quercetin	32.480	301.00	0.0113
11	trans-Cinnamic acid	32.364	147.00	0.0007
12	Kaempferol	32.473	285.00	0.3560
13	Naringenin	34.351	271.00	0.0368
14	Apigenin	34.930	269.00	0.0998
15	Luteolin	35.441	285.00	0.0061
16	Acacetin	40.949	283.00	0.0293

**Table 4:** The most favorable docking outcomes of *A. iva* phenolic constituents were docked with cyclooxygenase-2 (Protein Data Bank: 1CX2).

	Affinity Energy (Kcal·mol^−1^)	H-Bonds (Separation Å)	Hydrophobic Contacts	Additional Contacts
Native ligand (S58)	−9.4	Arg120 (2.32), Tyr355 (2.44), His90 (2.58), Gln192 (3.06)	Leu231, Ala527, Val349, Gly526, Tyr385, Leu384, Trp387, Leu352, Phe518, Val526	His90
Rosmarinic acid	−8.8	Gln192 (2.47), Ser353 (2.33) Tyr355 (2.65), Tyr385 (2.36), Leu384 (2.55)	Val523, Ser353, Gly526	Arg120
Rutin	−8.7	Val523 (2.22), Ser353 (3.07), Leu352 (2.26), Leu352 (2.23), Phe518 (2.45), Phe518 (2.04), Gln192 (2.93), Gln192 (2.73), Gln192 (2.51), His90 (2.08)	Phe518, Pro514	-
Apigenin-7-o-glucoside	−8.5	Tyr385 (2.62, 2.54), Gln192, Leu352 (3.10, 2.32, 2.85), Ser353 (2.64, 2.48), His90 (2.57), Tyr355 (2.33)	Leu352, Gly526, Ala527, Ser530, Val349, Leu359, Leu531	-
Acacetin	−8.3	Gln192 (2.82), Ser353 (2.65), His90 (2.49), Tyr355 (2.06), Leu384 (3.25, 2.39)	Ser353, Ala527, Val523, Leu352, Gly526, Leu526, Leu384, Tyr385, Trp387	-
Luteolin-7-o-glucoside	−7.6	Ser530 (2.60), Gln192 (2.52), His90 (2.71), Arg120 (2.35), Val116 (2.42)	Ala527, Val349, Leu352, Val523, Ser353	-
Naringenin	−7.5	Tyr355 (2.24), Val523 (2.40)	Leu352, Gly526, Ser353, Val355	Met522, Arg120
Kampherol	−7.1	His90 (2.65), Val523 (2.47), Ser530 (2.82)	Ala527, Val349, Leu531, Val523, Ser353	-
Apigenine	−6.7	Gln192 (2.59), His90 (2.75), Tyr355 (2.81)	Ser353, Ala527, Val523, Leu352, Gly526	Tyr385
Luteolin	−6.5	Leu384 (2.54), His90 (3.08)	Leu352, Gly526, Ser353, Val523, Val349, Ala527	-
Quercetin	−6.2	Ser353 (3.23), Met522 (2.82)	Gly526, Ala527, Leu527, Leu531, Val349, Leu352	Tyr385, Arg120

## Data Availability

Data supporting the findings of this study are available from the corresponding author upon reasonable request.

## Supplementary Materials

The following supporting information can be downloaded at: https://www.mdpi.com/article/10.3390/molecules31030496/s1, Figure S1: Two-dimensional (2D) chemical structures of the top bioactive metabolites identified from *Ajuga iva* extract (rosmarinic acid, rutin, apigenin-7-o-2 glucoside, acacetin, and luteolin-7-o-glucoside), selected based on their predicted binding affinities toward the cyclooxygenase-2 (COX-2) enzyme; Figure S2: Two-dimensional (2D) chemical structures of the top bioactive metabolites identified from *A. iva* extract (naringenin, kampherol, apigenine, luteolin, and quercetin), selected based on their predicted binding affinities toward the cyclooxygenase-2 (COX-2) enzyme.
