# Supplementary material for: Bioactive Potential and COX-2 Interaction of Ajuga iva (L.) Schreb. Hydroalcoholic Extract: Evidence from Experimental and Computational Studies
Source: Molecules. 2026 Jan 31;31(3):496. doi: 10.3390/molecules31030496 (PMC12898832; doi:10.3390/molecules31030496)
Supplement: Supplementary file 1 [file molecules-31-00496-s001.zip › molecules-4045152-supplementary.pdf]

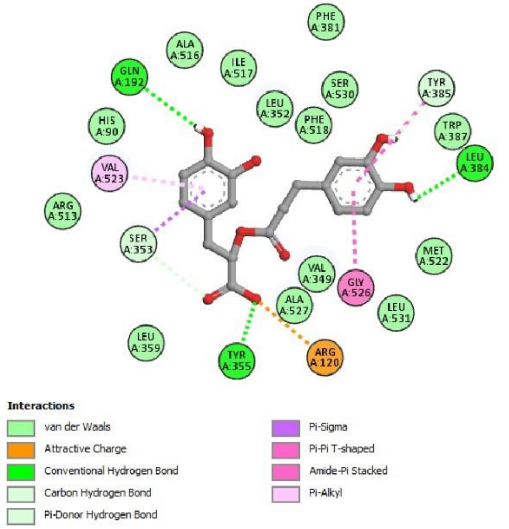

Rosmarinic acid

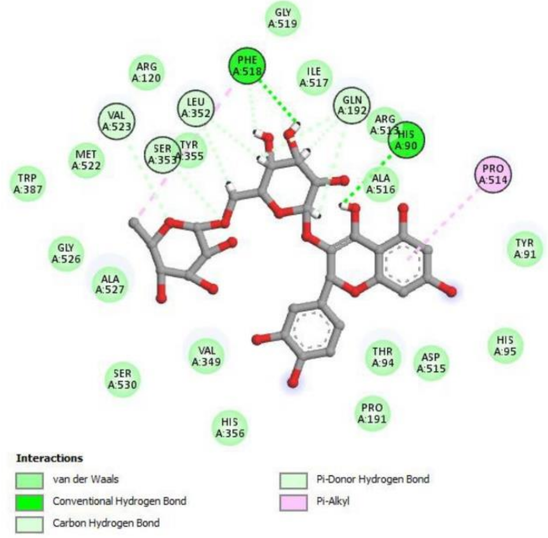

Rutin

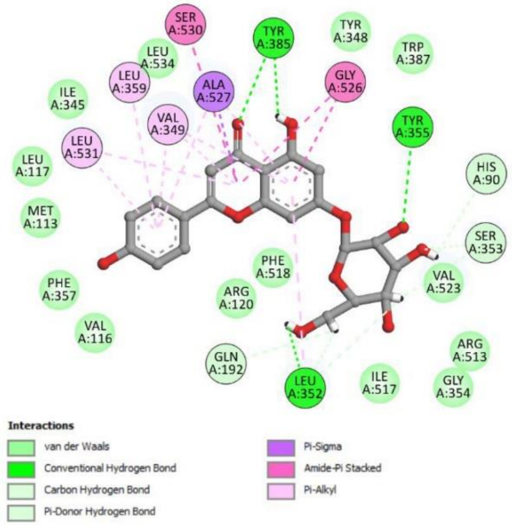

Apigenin-7-o-glucoside

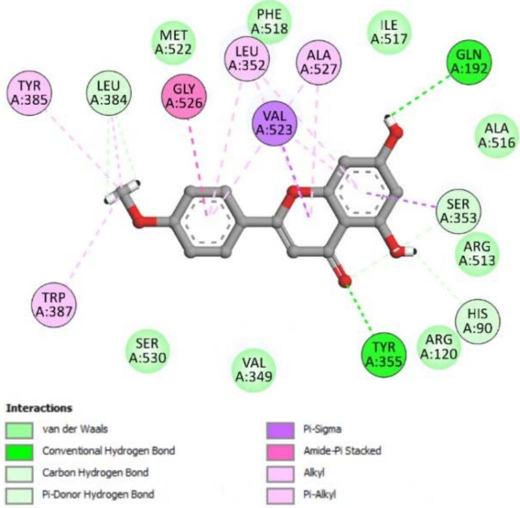

Acacetin

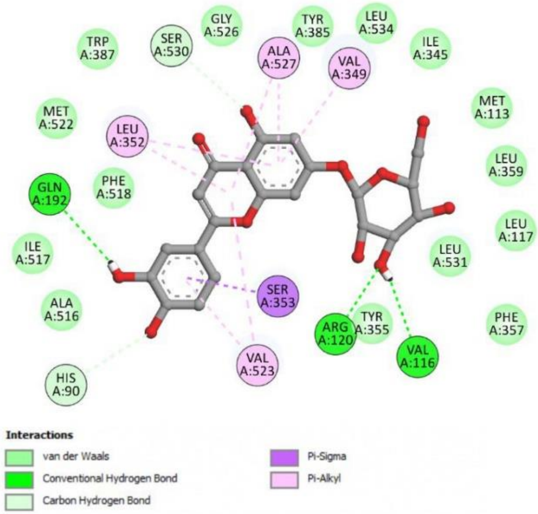

Luteolin-7-o-glucoside

Figure S1. Two-dimensional (2D) chemical structures of the top bioactive metabolites identified from *Ajuga iva* extract (rosmarinic acid, rutin, apigenin-7-o-glucoside, acacetin, and luteolin-7-o-glucoside), selected based on their predicted binding affinities toward the cyclooxygenase-2 (COX-2) enzyme

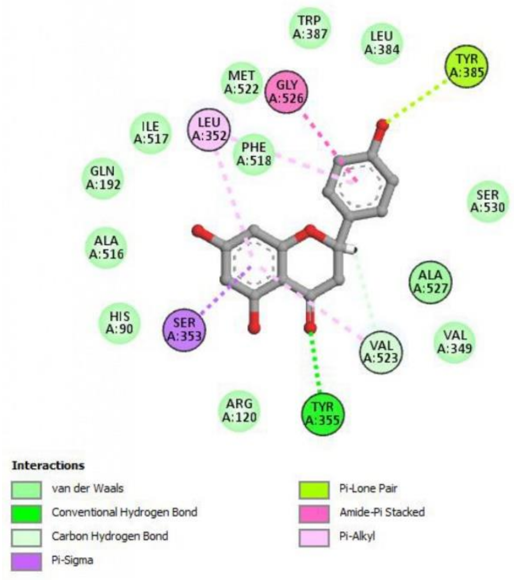

Naringenin

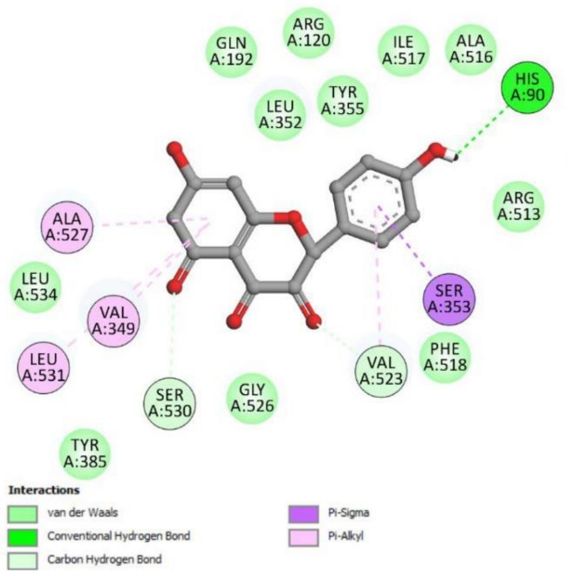

Kampherol

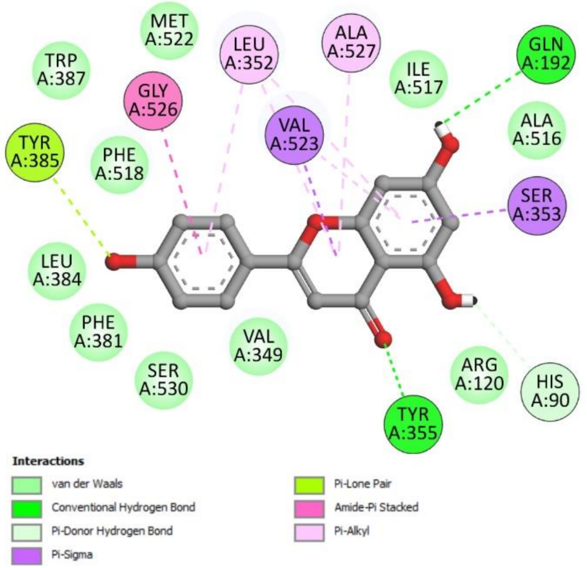

Apigenine

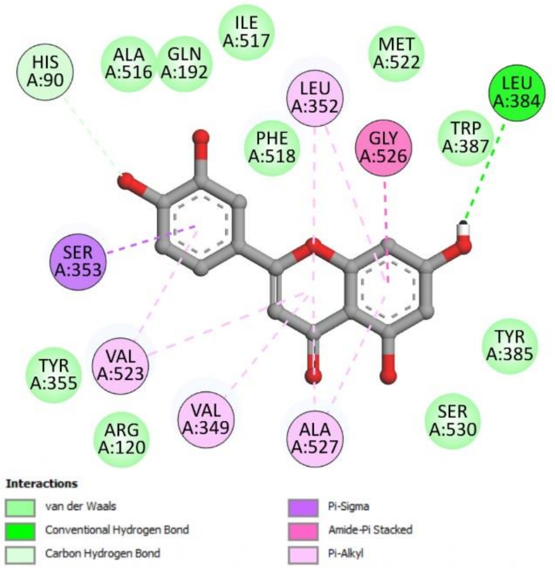

Luteolin

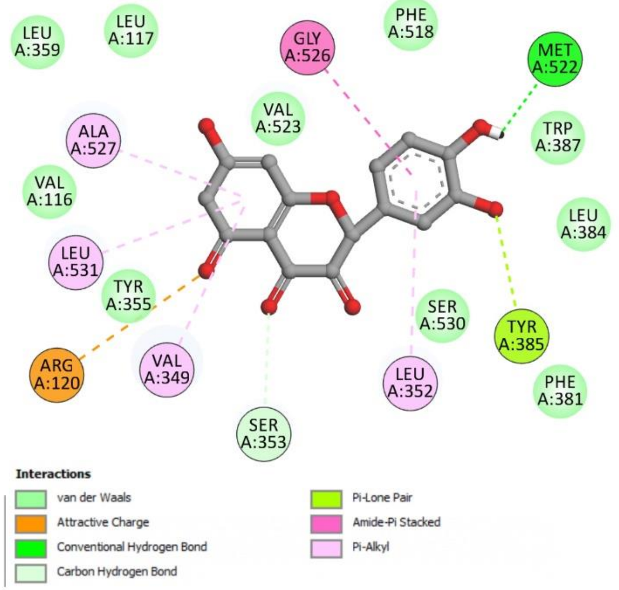

Quercetin

Figure S2. Two-dimensional (2D) chemical structures of the top bioactive metabolites identified from *A. iva* extract (naringenin, kampherol, apigenine, luteolin, and quercetin), selected based on their predicted binding affinities toward the cyclooxygenase-2 (COX-2) enzyme.
